# Unraveling the Connection between Fibroblast Growth Factor and Bone Morphogenetic Protein Signaling

**DOI:** 10.3390/ijms19103220

**Published:** 2018-10-18

**Authors:** Anna Schliermann, Joachim Nickel

**Affiliations:** 1 Lehrstuhl für Tissue Engineering und Regenerative Medizin, Universitätsklinikum Würzburg, Röntgenring 11, 97222 Würzburg, Germany; Anna.Schliermann@uni-wuerzburg.de; 2 Fraunhofer Institut für Silicatforschung, Translationszentrum TLZ-RT, Röntgenring 11, 97222 Würzburg, Germany

**Keywords:** bone morphogenetic protein, fibroblast growth factor, signal transduction, cross-talk, signal integration

## Abstract

Ontogeny of higher organisms as well the regulation of tissue homeostasis in adult individuals requires a fine-balanced interplay of regulating factors that individually trigger the fate of particular cells to either stay undifferentiated or to differentiate towards distinct tissue specific lineages. In some cases, these factors act synergistically to promote certain cellular responses, whereas in other tissues the same factors antagonize each other. However, the molecular basis of this obvious dual signaling activity is still only poorly understood. Bone morphogenetic proteins (BMPs) and fibroblast growth factors (FGFs) are two major signal protein families that have a lot in common: They are both highly preserved between different species, involved in essential cellular functions, and their ligands vastly outnumber their receptors, making extensive signal regulation necessary. In this review we discuss where and how BMP and FGF signaling cross paths. The compiled data reflect that both factors synchronously act in many tissues, and that antagonism and synergism both exist in a context-dependent manner. Therefore, by challenging a generalization of the connection between these two pathways a new chapter in BMP FGF signaling research will be introduced.

## 1. Introduction

Canonical signaling cascades activated by bone morphogenetic proteins (BMPs) and fibroblast growth factors (FGFs) both play crucial parts in essential cell and tissue functions, from early development to repair, maintenance and homeostasis of adult tissue. Hence it is not very surprising that BMPs and FGFs mechanistically interact with each other, and that their mostly divergent roles often result in a functional antagonism. Investigating the BMP-FGF interplay hence touches on many areas of interest in research, and insights into the underlying molecular interactions may represent fundamental biological processes in complex organisms.

The main focus of this review is on the inter-connections between BMP and FGF signaling. Their individual properties and their regulation will only be introduced shortly, as they have already been described in great detail elsewhere. (For reviews on FGF signaling, see References [1,2].) For a broad overview on BMP signaling, see Cytokine and Growth Factor Reviews’ special issues on BMPs, volumes 20 and 27).

## 2. Bone Morphogenetic Proteins

### 2.1. The Baffling Promiscuity in BMP Signaling

BMPs and growth and differentiation factors (GDFs) are dimeric growth factors forming a subgroup of the transforming growth factor β (TGFβ) family, with other subgroups, such as the eponymous “true” TGFβs (isotypes 1–3), the activins, and others like anti muellerian hormon (AMH) and GDF15 [3]. All TGFβ family members signal through heteromeric receptor complex assemblies, usually comprised of two type I and two type II serine/threonine kinase receptors [4]. An activated signal-receptor complex recruits intracellular signaling components at the type I receptors, most prominently receptor-regulated so-called (R-)SMAD (abbreviation derived from homologies to *Caenorhabditis elegans* SMA (“small” worm phenotype) and Drosophila MAD (“Mothers Against Decapentaplegic” proteins) proteins. R-SMADs belong to one of two subgroups, and each type I receptor (with the exception of activin-like kinase 1 (ALK1)) activates SMADS of just one subgroup: Most BMP/GDF receptors phosphorylate SMADs 1, 5 and 8, whereas the TGFβ type I (ALK5) and the activin type I receptors phosphorylate SMADs 2 and 3. Activated R-SMADs form heterotrimers with one so-called common-mediator (Co-SMAD) SMAD4 protein, and translocate into the nucleus to exert their influence on gene expression with the help of co-factors and histone modifying enzymes. Apart from this canonical signaling pathway, BMP and TGFβ receptors have also been reported to activate other signaling proteins, including mitogen-activated protein kinases (MAPKs), such as p38 and Erk, as well as phosphoinositide 3-kinase (PI3K) [5,6,7].

### 2.2. BMP Signal Regulation

It appears plausible that a signaling system with high receptor promiscuity and limited downstream events like this one can only generate so many distinct functions if it is extensively regulated. Diverse regulatory mechanisms exist at any point in the signaling cascade. Intrinsically, receptor specificity and ligand concentration determine, such as active signaling complexes can form. A multitude of extracellular modulators, e.g., Noggin, Follistatin, Chordin, and others, prevent receptor-ligand interaction by sequestering ligands and masking their receptor-binding epitopes [6,8], and some extracellular matrix components like heparin or heparan sulfates, but also pseudo-receptors like BAMBI may likewise deflect BMP ligands from their receptors [6]. Intracellularly, R-SMAD phosphorylation can either be stabilized (cyclin-dependent kinase (CDK) 8 and CDK 9) or destabilized (SMURF proteins, inhibitory (I)-SMADs (SMAD 6 and 7)), influencing the duration of the active signal [4,5,9,10]. Receptor stability on the cell surface, and SMAD entrance into the nucleus are also being regulated (e.g., MAN1, [11]), and the influence of phosphorylated R-SMADs on transcription is dependent on the presence or absence of co-factors and/or co-repressors [4,7,8,9].

## 3. Fibroblast Growth Factors

### 3.1. An Omnipresent Growth Factor System

In the adult organism, FGF signaling is active in most tissues and fulfills vital functions both in the cellular and in the tissue metabolism. Looking at the level of individual cells, FGFs are linked to proliferation, migration, and/or differentiation, which translates into the regulation of tissue homeostasis and repair [1]. These multiple roles and the vast presence of active FGF signaling become evident in the high number of ligands, receptor splice variants, and downstream effectors of this signaling system.

The FGF family comprises 22 members, 18 of which get secreted and are able to bind to one or more of the four FGF receptors (FGFR) 1–4. FGF ligands are monomeric factors whose close association to extracellular matrix components, in particular to heparan sulfate proteoglycans (HSPGs), determine their auto- or paracrine nature. HSPGs not only limit the distribution of FGFs, but also act as stabilizers of ligand-binding to the respective receptors [2]. Only three members of the FGF ligand family have a reduced affinity to HPSGs and hence function as endocrine factors (FGF 19, -21, and -23). They signal via the same four FGF receptors, but utilize Klotho proteins instead of HPSGs as co-receptors [1].

FGF receptors are tyrosine kinases with a single transmembrane domain that dimerize and become autophosphorylated upon ligand binding. They exist as various isoforms derived from alternative splicing events, adding further complexity to the FGF signaling system. Once activated, FGFRs trigger four main pathways within the cell, comprising mitogen-activated protein (MAP) kinases, phosphoinositide 3-kinase (PI3K), signal transducers and activators of transcription (STATs) and phospholipase Cγ (PLCγ). The adaptor protein FGFR substrate 2α (FRS2α) directly binds to the FGFR and triggers signaling via MAP kinase and PI3K cascades via growth factor receptor-bound protein 2 (GRB2) [2,12]. STATs 1, 3, and 5, as well as the PLCγ pathway do not utilize any adapter proteins, but are activated upon direct binding to the phosphorylated FGFR. STAT proteins, as well as Akt and MAP kinases (including ERK1/2, p38 and JNK) translocate into the nucleus to alter the transcription of target genes once they are phosphorylated. PLCγ influences the calcium ion levels within the cell and activates protein kinase C (PKC) [1].

### 3.2. FGF Signal Regulation

As is the case in BMP signaling, various growth factor functions are governed via a small number of receptors. Hence, signal transduction has to be tightly and variably regulated. At the cell surface, the system gains plasticity by the high number of different FGF ligands, alternative splicing and glycosylation status of the FGF receptors, and the necessity for co-receptors, such as HPSGs or Klotho proteins, as has been mentioned above. FGF ligands also experience short half-lifes, due to free cysteines. Consequently, not every cell capable of FGF signaling can respond to every ligand, and signaling becomes cell-type specific [1,13,14]. Receptor internalization is then able to limit the signal duration [2], at least if the receptor is subsequently degraded. Otherwise, the signaling may continue from within the cell, independently of extracellular ligand changes [13,15].

Intracellularly, most regulatory proteins only address one or two of the four activated pathways. For instance, binding of GRB14 to the activated FGFR1 only inhibits the activation of PLCγ. If in turn FRS2α and/or GRB2 are targeted, for example by ubiquitination via CBL (an E3 ubiquitin ligase) or by dephosphorylation via Src homology region 2-containing protein tyrosine phosphatase 2 (SHP2), only the MAP and PI3 kinase pathways are impaired [1]. However, CBL can also directly ubiquitinate the receptor to initiate its internalization, effectively terminating signaling by this receptor [2]. The tyrosine kinase inhibitor Sprouty (SPRY), which not only inhibits FGFRs, but also VEGFRs, PDGFR and NGFR, interacts with GRB2 and likewise affects signaling linked to this adapter protein, albeit with a prevalence for MAPK inhibition. Downstream, transmembranic SEF (similar expression to fgf genes) binds to the MEK-MAPK complex, inhibiting only the MAPK pathway. Finally, MAPK phosphatases can terminate signaling of individual MAP kinases, thus blocking only parts of the MAP kinase route. For example, MKP3 is specific for Erk kinases and therefore does not influence an active JNK or p38 signal [1]. ERK in turn is able to limit FGFR signaling more comprehensively by phosphorylating FRS2α and thus inhibiting GRB2 binding [2].

### 3.3. The Curse of Promiscuity and Omnipresence

Both BMP and FGF signaling systems contain a number of ligands that by far exceeds the number of their receptors, and both fulfill multiple functions in various tissues. Subsequently, both systems need to be extensively regulated and fine-tuned by distinct spatial and temporal expression patterns and a variety of regulatory proteins, as has been briefly addressed above.

**Figure 1:**
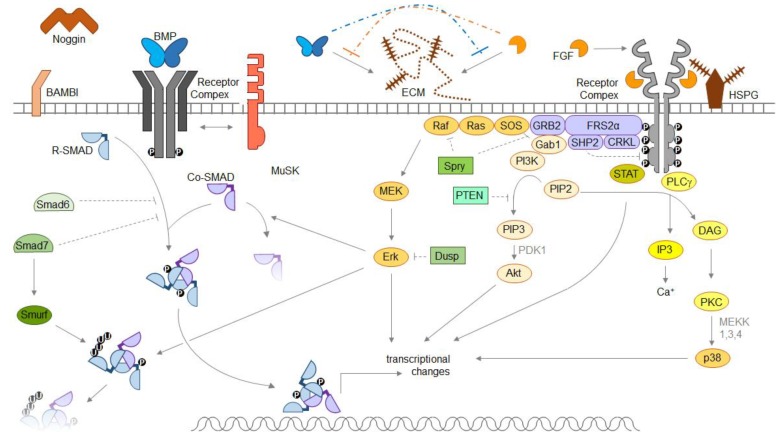
Molecular mechanisms of fibroblast growth factor (FGF)-bone morphogenetic protein (BMP) interactions. Depicted are canonical BMP- and FGF signal transduction pathways. BMPs are dimeric proteins, assembling heterotetrameric receptor complexes and activating R-SMAD proteins to translocate into the nucleus and influence transcription. Extracellular inhibitors like Noggin, membrane-associated inhibitors like BAMBI or intracellular inhibitors like Smurfs limit BMP signal transduction. FGFs recruit two FGF receptors and heparan sulfates into the signaling complex, and activate multiple-step cascades with effector kinases, including Erk, Akt and p38. The muscle-specific kinase MuSK is able to influence BMP signaling via binding to BMP ligands without involvement of its own kinase domain. Abbreviations: BMP—bone morphogenetic protein; Co-SMAD—common mediator SMAD; CRKL—Crk-like protein; DAG—diacylglycerol; Dusp—dual specificity phosphatase; ECM—extracellular matrix; FGF—fibroblastic growth factor; FRS2α—FGFR substrate 2α; Gab1—GRB2-associated-binding protein 1; GRB2—growth factor receptor-bound protein 2; HSPG—heparan sulfate proteoglycan; IP3—inositol triphosphate; MAPK—mitogen-activated kinase; MEK—MAPK kinase; MuSK—muscle-specific kinase; PDK1—pyruvate dehydrogenase lipoamide kinase isozyme 1; PI3K—phosphoinositide 3-kinase; PKC—protein kinase C; PIP2—phosphatidylinositol 4,5-biphosphate; PIP3—phosphatidylinositol 3,4,5-trisphosphate; PLCγ—phospholipase Cγ; PTEN—phosphatase and tensin homolog; R-SMAD—receptor-regulated SMAD; SHP2 – Src homology region 2-containing protein tyrosine phosphatase 2; Smurf—SMAD specific E3 ubiquitination regulatory factor; SOS—Son of sevenless; Spry—Sprouty; STAT—signal transducers and activators of transcription.

A graphical depiction of general pathway components of both pathways is shown below (Figure 1).

We must remain aware that any biological effect observed is only the sum product of a whole concert of interaction partners, and that connections between single events within one of these pathways are not always as straightforward as we would like them to be. Receptor hyper-activation does not necessarily lead to an elevated biological response to its ligand [13]; highly activated intracellular signaling does not necessarily rely on abundant receptor levels on the cell surface [15]; ligand synergism in one cellular context does not necessarily exclude antagonism in the next [16]; receptor activation does not necessarily lead to downstream signaling [16]; and boosting receptor affinity in a ligand does not necessarily render it a more potent agent [16]. While it is certainly out of scope for any publication to address all possible ways of regulating the respective ligand-receptor interaction in focus, it is worth keeping in mind that any causal link made between two events within one of these signaling pathways is a context-dependent connection and subject to re-evaluation under different circumstances.

## 4. The Cross-Talk between BMP and FGF

### 4.1. Development

Cell fate determination and axis formation are often the result of growth factor gradient formation, or the integration of different gradients, of morphogens, such as BMPs and FGFs. In this context, BMP-FGF antagonism has been described as “constituting a common module” [17], and examples can be found both in axis formation, as well as in tissue specification [17].

The central roles of TGFβ family members and FGFs in early development already become evident at the very first stages of cell fate determination. For example, once the fertilized egg develops into a hollow sphere (the blastocyst), the inner cell mass ICM (which gives rise to the embryo itself) can be distinguished from the outer cell layer (trophoblast). Pluripotency factors Oct4 and Nanog within the ICM induce FGF4 expression to elevate FGFR2-mediated MAPK signaling, which in turn leads to the ICM’s separation into the epiblast (expressing Nanog and FGF4) and the primitive endoderm (expressing FGFR2 and its downstream target *Gata6*, a Nanog-suppressor) [18]. The epiblast represents the origin of the three germ layers. At its posterior, the primitive streak (mesendoderm) is associated with the expression of Wnt3, Brachyury, as well as the TGFβ family member Nodal, while the anterior part of the epiblast develops into epidermal tissues [19]. FGF8-activated FGFR1 facilitates the continued expression of Brachyury and *Tbx6*, as well as a high migratory potential in descendants of the primitive streak and thereby determines mesoderm, the origin of the musculoskeletal system and others [20].

Apart from the three germ layers, the early embryo also develops three distinct axes, the anterioposterior (AP) one along which the germ layers originally form, the dorsoventral (DV) axis, and the left-right (L/R) axis. FGF signaling (most prominently the FGFs 4 and 8) and TGFβ/BMP signaling (mainly Nodal, as well as the BMPs 2, 4 and 7) are involved in the formation of all three axes: Along the AP axis, extraembryonal BMP2/4 signaling via the type I receptor BMPRIA helps to determine the posterior primitive streak within the embryonal tissue [17,21]. The primitive streak expresses Nodal, with high Nodal levels determining endoderm, and medium Nodal levels together with FGF8/FGFR1 signaling drive mesoderm determination, as stated above [19,22]. During the subsequent AP separation into the individual germ layers, FGFs show strong posteriorizing capacities along with Wnt and retinoid acid [22]. Along the DV axis, Wnt initiates dorsal cell fates, whereas BMPs are strongly ventralizing. Here, Wnt-induced FGFs antagonize BMPs on several levels: By BMP expression downregulation, Chordin and Noggin upregulation, and SMAD inactivation via Erk mediated tyrosine phosphorylation [17,22]. Finally, the L/R axis is predominantly determined by the antagonism of Nodal (left) and Lefty1 and 2 (right), but BMPs and FGFs are still involved in the early steps of L/R axis formation, as well as in the regulation of Nodal expression [17,21].

### 4.2. Bone and Cartilage

The appendicular skeleton. The skeleton forms mainly from mesodermal tissue, although parts of the skull and teeth originate from ectodermal precursors. Signaling events in osteochondral development has been most extensively studied in the context of appendicular skeleton formation, which starts with the appearance of limb buds in the lateral plate mesoderm. Their outgrowth is driven by FGFs (mainly FGF8, but also FGF4 and FGF17) and Gremlin-1, a BMP inhibitor. Distally, expression of BMPs signaling via BMPRIA (including BMPs 2, 4 and 7) limits FGF signaling and limb bud outgrowth [20,23,24,25].

Histologically, the formation of limb bones is described by a number of steps in a process called endochondral ossification: (1) Mesenchymal cell condensation, (2) chondrocyte proliferation, (3) column formation (pre-hypertrophy), (4) hypertrophy, and (5) vascularization and mineralization. While most chondrocytes in the skeletal anlagen become hypertrophic and are replaced by bone forming cells, there are two regions where cartilage persists after birth: The epiphysis or growth plate, whose chondrogenic core keeps proliferating and undergoing hypertrophy for the sake of longitudinal growth until early adulthood and the joints, where non-proliferative cartilage resides on top of the bones, flanking the joint space, avoiding hypertrophy through life [23]. 

Understandably, the molecular regulatory processes governing these steps are quite complex, including the influence of a multitude of growth factors, such as BMPs and FGFs, as well as Ihh, Wnt ligands, parathyroid hormone related protein PTHrP, and TGFβs; which regulate key transcription factors like Sox9 (cartilage) and Runx2 (bone), as well as various homeobox genes [26,27,28]. Seemingly, every knot of this signaling network is connected to every other: TGFβ influences PTHrP, which influences hedgehog proteins, which influence BMPs, which influence FGFs, which influence Wnts, which influence TGFβs, etc. [28,29,30,31]. Importantly, the inter-connection of BMP and FGF signaling in endochondral ossification entails synergistic and antagonistic parts: For example, BMPs (mainly BMP2 and BMP4, but also TGFβs) promote chondrocyte differentiation and proliferation, while FGFs (mainly via FGFR3) inhibit these processes. This has been exemplified by studies showing that severe forms of chondrodysplasia can either be caused by down-regulation of BMP2/BMPRIA, BMPRIB signaling [32] or by up-regulation of FGFR3 signaling, respectively [2,33]. This antagonism is mediated, at least to some extent, via opposing regulation of the chondrocyte-inducer and -stabilizer Sox9, and via negatively influencing each other’s expression levels [23,27,29,33,34]. One step further in the course of bone formation, hypertrophy and osteoblast differentiation are mediated by the transcription factor Runx2, a Sox9 antagonist [34,35,36]. TGFβ ligands and SMAD2/3 signaling actually inhibit Runx2 function and thus prevent chondrocyte hypertrophy, while BMP ligands (especially BMP2 and BMP6, which are highly expressed in hypertrophic zones [33]) and SMAD1/5/8 signaling strongly promote hypertrophy [26,34,37,38]. BMP2 induces the translocation of Runx2 into the nucleus, and it has been shown that FGF2 is actually necessary for this induction [28,39]. Further indications for the positive effect of both FGF and BMP signaling on cell hypertrophy can be seen in the phenotypes of FGF9 KO or FGFR1 cKO mice driven by the *Twist2* promoter, which is active in early osteoblastic stages: Both gene deletions lead to the same phenotype, namely reduced hypertrophic zones [33]. In contrast to the proliferation zone, in which only FGFR3 is expressed, pro-hypertrophic FGF signaling seems to be transmitted via FGFRs 1 and 2, which are expressed in or adjacent to hypertrophic zones [36]. Thus, in contrast to the mechanisms stated for chondrocyte proliferation, BMPs and FGFs closely synergize to promote chondrocyte hypertrophy and osteoblastic differentiation, both by positively regulating the expression of each other’s signal cascade components, as well as by synergistically increasing the expression and thus activity of Runx2 [28,29,39]. Notably, while the BMPs involved seem to be mainly the same in the two processes; the involved FGF receptors differ.

Cranium. Some parts of the skull, including but not limited to the cranial base, develop via endochondral ossification as well. These structures are paraxial mesoderm-derived and are collectively referred to as the chondrocranium. As in long bones, there are cartilage sites that persist after birth to function in the same way as growth plates, called synchondroses, and the steps of bone formation, as well as the molecular regulation is highly similar to long bone and growth plate mechanisms [33,40]. 

Skull parts forming via membraneous ossification are neural crest-derived. They encompass the facial bones (viscerocranium) and the membraneous neurocranium, entailing most of the calvaria [40]. To allow for growth and expansion of the calvaria after birth, membraneous bones of the skull are not divided by chondrogenic synchondroses, but by fibrous connective tissue, the sutures.

The mechanisms involved in membraneous ossification, suture formation, and suture maintenance are quite similar if compared to later stages of endochondral ossification: For example, Runx2 is an essential driver of osteoblast differentiation in membraneous ossification as well [41], and mesenchymal proliferation is likewise regulated by TGFβ ligands, Wnt and FGF signaling [40]. On the other hand, Sox9 haplo-insufficiency does not cause malformation of the cranial skeleton [41], suggesting a far less important role for Sox9 in cranial development. This is not surprising, as it is the regulator of chondrocyte differentiation, a developmental step absent in membraneous ossification. Likewise, Ihh and PTHrP, regulators of chondrocyte proliferation and pre-hypertrophy, seem to be less involved in membraneous ossification as well. Their mutations seldomly yield cranial phenotypes, although they are present in the developing calvaria [36,42,43]. It seems that while the players in membraneous and osteochondral ossification are mainly the same, their importance within the signaling network and their impact on the physiological development shifts depending on the type of bone formation.

### 4.3. Other Tissues

As BMPs and FGFs are quite ubiquitously expressed and involved in essential cell functions, such as proliferation and differentiation, they meet in many other tissues as well. Skeletal development, as described above, is a very prominent example, but far from being the only one.

Teeth, another mineralized tissue, develop by close interaction of the oral epithelium and the underlying neural crest-derived mesenchyme. The integration of its four core signaling pathways, Wnts, hedgehogs, BMPs (mainly 2 and 4), and FGFs (including 3, 4, 8–10, depending on the expressing cellular subtype and particular function), is largely independent of the surrounding tissues [44]. Due to the high connectivity of this signaling network, synergistic and antagonistic relationships between FGFs and BMPs are hard to distinguish [45]: BMPs and FGFs antagonize each other in the regulation of tooth type-determining homeobox genes [46], while positively influencing each other’s expression via some of the same genes: For example, Pax9, a key regulator of tooth development, is up-regulated by FGF8, subsequently induces BMP4 expression, which in turn suppresses it again [45]. While FGF8 and BMP4 are clearly antagonistic in terms of patterning the border between incisors and molars, they both induce and can be maintained by Msx1, another key transcription factor [45,47]. On the level of a single tooth anlage, enamel knots (an organisatory unit determining tooth shape and dental cell differentiation) express FGFs (including 4 and 9), as well as BMP4, but no FGF receptors. Hence, the FGFs do not act on the enamel knots themselves, but initiate the proliferation of surrounding cells, and enamel knots eventually undergo BMP4-driven apoptosis [46,47]. 

Eyes and Skin. Other ectodermal tissues, such as skin and eyes, likewise rely on FGF-BMP interaction in their development. For example, the optical lens is induced both by FGF and BMP7 signaling [48] and both signals, mediated via FGFR1/2 and BMPRIA/IB, respectively, have positive effects on lens fiber cell differentiation [49,50]. BMPs and FGFs are also involved in retinal cell fate determination [51], although here, their antagonism defines the tissue border between neural retina (FGF8 via FGFR3) and pigmented epithelium (BMPs 4 and 5) [52,53]. 

Skin originates from ectodermal progenitors due to (Wnt-mediated) BMP4 signaling in antagonism with the FGFs that are linked to neural development. While both BMPs and FGFs continue to be expressed at the sites of epidermal and dermal development, they are not described as key players in skin maturation or skin cell differentiation [54,55]. 

Central Nervous System. For ectodermal progenitors, neural differentiation is the alternative route to epidermal differentiation, and according to the default model, it is largely driven by the absence of BMP4 signaling. This is enforced by a number of BMP inhibitors, such as Noggin, Follistatin and Chordin, rather than by the presence of an inductive agent [56,57]. While it has been suggested that FGF signaling (most prominently FGF8) also antagonizes BMPs and thereby triggers neuralization [54,58], it seems more likely that it is involved in embryonic stem cell priming towards an epiblast-like state, meaning involvement already at the beginning of germ layer definition, and that it should be considered a pre-requisite for subsequent neural differentiation rather than a direct neural inducer [56,59]. Once neural fate is determined, the anterior-posterior and dorso-ventral organization of these CNS cells is patterned not only by BMPs and BMP inhibitors, but by FGFs, hedgehogs, Wnts, and retinoid acid as well [56,60]. Here, BMP-FGF interaction is usually antagonistic, as can be seen for example in the FGF-dependent survival of telencephalic precursors blocking SMAD2/3-mediated apoptosis [61], in border formation between the telencephalic dorsal midline (BMP4) and cerebral cortex (FGF8) [62], or in the development of posterior neural tissues (hindbrain and spinal cord), where BMP inhibitor levels expressed by the anterior organizer are low, and FGFs, including FGF3, take over to antagonize BMPRIA-mediated, ventralizing signals intracellularly, allowing neural differentiation and specifying posterior cell fates [63].

Kidneys and Heart. On the mesodermal side, FGF-BMP interaction can be observed for example in nephrogenesis and cardiogenesis. In nephrogenesis, once the uteretic bud forms from the intermediate mesoderm, bud outgrowth and branching is initiated by FGF7 and FGF10 from the adjacent mesenchyme, signaling via FGFR2 expressed in the outgrowing tip. BMP4 is one of the negative regulators of this outgrowth. Down the road of kidney development, other members of these growth factor families, namely BMP7, FGF9 and FGF10 synergize in promoting the maintenance and proliferation of nephron progenitors [64]. In the development of cardial tissue, mesoderm is specified and subtype patterning is achieved by the combination of Nodal and FGF8 signaling in the presence of BMP inhibitors, such as Chordin, Noggin, and Follistatin. Once the visceral mesoderm is specified, BMP2 and 4 expressed by the adjacent endoderm now synergize with FGF8 and 4 signaling to induce heart-specific markers. BMP2/4 continues to drive precursor differentiation, for example in myofibrillogenesis, while FGFs, including FGF9, continue to be involved in cardiac cell proliferation [65,66,67].

Lung and Gut. The gastrointestinal tract, encompassing thyroid, stomach, intestine, lung, liver, and pancreas, develops along the AP axis in close communication between mesoderm and endoderm. Among the key factors, FGFs and BMPs, expressed in the mesoderm, help to determine the endodermal cell fates along the axis [68]. For example, FGFs 1, 2 and 10 initially synergize with BMP4 in fate determination of pulmonal precursors from the foregut epithelium. However, just as in kidney development, BMP4 and FGF10 signals via FGFR2IIIb then antagonize in the regulation of lung bud outgrowth and branching. As FGF signaling enhances the expression of its antagonist BMP4 itself, it self-limits its proliferative potential at the bud tip, while still stimulating peripheral outgrowth, causing branching. FGF-induced Spry expression limits the overall outgrowth [69]. In other regions of the foregut, Wnt and FGF4 suppression enables liver and pancreas induction. Once liver- and pancreas-forming domains are specified, mesodermally expressed FGF1, FGF2 and BMP4 synergize to favor hepatic over pancreatic cell fates [70]. In the mid- and hindgut regions, Wnt and FGF4 signals are active and intestinal cell fates are adapted [71]. BMPs and FGFs resume an antagonistic relationship in the intestinal epithelium, where BMPRIA-driven signaling and FGFR3-driven signaling differentially regulate Wnt in the formation and maintenance of stem cells in crypts [72,73].

### 4.4. Similarities and Discrepancies in BMP or FGF Related Disorders

In order to investigate FGF- and/or BMP-related disorders, knock-in or knock-out models provide the basis to understand the specific role of these factors in the native developmental processes, as well as in maintenance of any given tissue.

Mutations in FGF ligands and receptors affect a plethora of tissues in both mice and men. Due to the pleiotropic functions of FGFs, mutations in a single FGF family member can have widely differing consequences, ranging from early embryonic lethality over (severe) neuronal or cardiac deficiencies to short legs or long hair [1]. In terms of FGF receptors, germinal gain-of-function mutations usually cause chondrocyte and osteoblast dysfunctions, most prominently dwarfism and craniosynostosis [74], while somatic gain-of-function mutations are associated with elevated proliferation and survival of cells, hence cancer [2].

In contrast to FGF signaling derailing, mutations in BMP signaling components rarely affect cancer progression, as their physiological functions are mostly linked to differentiation processes or apoptosis, rather than proliferation or cell motility. A vast literature search by Skovrlj et al. yielded no evidence of BMP2 causing tumors de novo [75], although both tumor-promoting and tumor-suppressing actions of BMPs have been reported in general, dependent on the particular cellular context: BMPs seem to antagonize cancer proliferation and cancer stem cell survival, but to positively influence migration and angiogenesis [76,77]. Nonetheless, BMPs are usually considered tumor suppressors [78], and tumor promotion is linked to TGFβs rather than to BMPs [76,79].

Just like for FGFs, mutations in BMP ligands and their receptors have widely differing consequences, depending on the affected component and/or the specific mutation. Loss-of-function of central BMP ligands or receptors can result in (early) embryonic lethality, while non-lethal phenotypes range from severe and progressive disorders, such as pulmonary hypertension or fibrodysplasia ossificans progressiva, via eye malformations to irregularities in single finger joints [80].

The wide range of phenotypes caused by members either of the FGF or the BMP family is quite startling. Based on their omnipresence and developmental importance, one would assume that mutations that affect one of them should not be compatible with life (and for some, this is certainly true). However, the reason why most mutations in a single ligand or receptor cause non-lethal phenotypes or syndromes lies in their vast promiscuity and excessive regulation. These are also the reason why different mutations in the same protein can sometimes produce bafflingly distinct phenotypes. In FGFR3, a P250R mutation causes the closure of a specific cranial suture, sometimes accompanied by hand malformations and deafness, while an A391E mutation causes craniosynostosis accompanied by a skin condition, and a G380R mutation neither affects cranial sutures nor skin or hearing ability upon birth, but causes achondroplasia resulting in dwarfism (OMIM 134934). Even more discrete phenotypes arise upon specific mutations in the BMP ligand GDF5, sometimes only affecting one or few determined finger joints [81].

While we do not understand why some specific mutations cause such specific disorders, the focus on shared phenotypes between FGF and BMP family members nonetheless helps to shed light on their molecular connections.

### 4.5. Meeting of the Titans in Craniosynostosis

Syndromes rooted in either BMP of FGF signaling mutations are mostly exclusive in their phenotypes, but do sometimes overlap in bone/cartilage malformations, such as craniosynostosis.

Craniosynostosis is described to originate from enhanced bone formation rather than altered proliferation, as is the case in growth plate deficiencies [37,82]. While syndromic craniosynostoses caused by FGF receptors 1, 2 or 3 usually affect coronal or multiple sutures, the most commonly affected suture is the sagittal one (or both midline sutures), including in some syndromic forms that are not linked to FGF signaling [82,83,84]. By genotyping, midline suture fusions in non-syndromic craniosynostosis patients were most prominently linked to haplo-insufficiency of SMAD6, a BMP inhibitor [83]. Other enhancing mutations of BMP signaling, such as deficiency of the inhibitory SMURF1 or duplication of the downstream mediator Runx2, also cause the fusion of midline sutures during infancy [85,86].

In contrast to the midline sutures, the coronal suture is unique in its embryonic origin, emerging between the neural crest-derived frontal bone and mesodermal parietal bone. As mutations in the neural-crest related osteoinductive Twist1 or site-specific expression of a constitutive active BMP type I receptor (BMPRIA) in cranial neural crest both also cause premature suture closing [82,84,87], the pathomechanism may be dependent on the developmental background of the affected cells [83]. 

Witnessing the underlying mutations, it seems that both elevated BMP and/or elevated FGF signaling may cause premature suture closing. This indicates BMP-FGF synergism in the context of craniosynostosis. Indeed, it has been described that FGFR2 and FGF2 suppress the expression of the BMP inhibitor Noggin, whose expression is vital for patent cranial sutures. In turn, Noggin overexpression in skulls of healthy mice prevents natural suture fusion [88]; and application of ectopic recombinant Noggin rescues rats overexpressing FGFR2 from the craniosynostosis phenotype [89], as well as prevents the recurrence of suture closure after they need to be surgically opened [31]. As FGF2 has been described to suppress Noggin only at high concentrations [88] and to induce BMP2 expression in cranial suture cells [90], it seems that BMP activity is either permitted and/or supported only by these high FGF concentrations. Once this threshold is reached, FGF and BMP work together to drive osteoblast differentiation and mineralization, or suture closure, in an additive/synergistic manner. Both FGF and BMP signals appear to be essential for suture closure, as FGF-driven ossification appears quite BMP-dependent [31,90]. However, this interaction may be rather context-dependent, as not all pre-fusing sutures express higher BMP2/4 and lower Noggin levels [91], and as high doses of FGF2 have been described to lower BMP2 levels via the induction of Noggin at another side of bone formation [92]. Taken together, it appears evident that suture homeostasis is the result of a very specific composition of cells/tissues and a fine balance between synergistic FGF and BMP signaling events.

**Figure 2:**
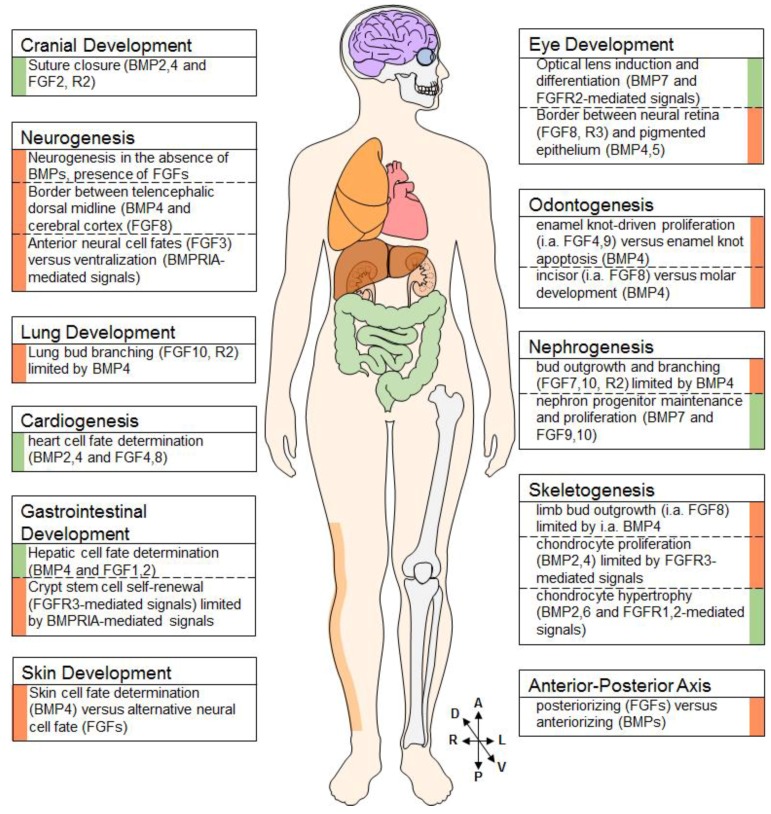
Examples of BMP-FGF Interaction throughout the Developing Body. BMPs and FGFs interact in (the development of) various tissues, such as the brain, lung, heart, gut, skin, eyes, teeth, kidneys, and the skeleton. Examples of a synergistic nature are flanked by a green indicator, antagonistic examples are flanked by a red indicator. (cartoon provided by www.motifolio.com).

The inter-connections between FGF and BMP signaling, which have been briefly mentioned in the preceded sections are summarized in Figure 2.

### 4.6. Connecting Two Signaling Pathways

With the BMP and FGF families having as many members and functions as they have, their molecular interaction with each other can hardly be generalized for all developmental stages and tissues. Most observations are context-dependent, and many examples of that have been detailed above. One is BMP2/FGF2 synergism in ossification, and underlying mechanisms entail influencing members of each other’s signaling pathway, as well as regulating shared target genes: BMP2 and FGF2 positively regulate each other’s expression in murine bone [29,39], and FGF2 can increase BMP receptor expression in sutures as well [90]. FGF2 and BMP2 both have a positive influence on Runx2 expression, and mice homogeneously lacking FGF2 show impaired nuclear accumulation of BMP2-induced Runx2. Applied research approaches try to exploit this by using both factors in grafts for bone repair [93,94,95], although the success may depend on both the sequence of their application, and their respective concentrations [92,93,96]. It appears that conditions have to be “just right” to allow synergism. Otherwise, antagonistic effects take over: In proliferating chondrocytes, BMP signaling inhibits FGFR1 expression [28], and FGFR3 activity in turn down-regulates expression of the BMP type I receptor BMPRIA [33]; FGF2 increases Noggin and SMAD6 expression during the formation of ectopic bone formation, thus antagonizing BMPs [92], and reduces BMPRIA or IB expression in the growth plate of mice correlates with elevated levels of FGFR1, FGF4, and FGF8 [20,97]. 

Only a few interactions have been encountered multiple times and can be considered more general: For example, Erk and other MAP kinases are known to phosphorylate SMAD1/5 proteins in their linker region, hindering their translocation into the nucleus and promoting their degradation. The kinases JNK, p38 and Erk may also cause proteosomal degradation of SMAD4 [8,98,99].

### 4.7. Cell Surface Integration

All examples described here have in common that they study BMP-FGF connections in terms of their effectors (e.g., SMADs or MAPKs), and/or in terms of transcriptional changes of selected genes. If and how BMPs and FGFs influence each other on the cell surface remains mainly hypothetical, although they share important binding partners in the extracellular matrix (ECM). Most FGFs and many (SMAD1/5/8-inducing) BMP ligands are paracrine factors because of their ECM association, and heparan sulfates are the mediators (see Figure 1).

Heparan sulfates (HS) represent a heterogeneous group of sulfated disaccharide chains, anchored to the cell membrane or the extracellular matrix (ECM) (or, in some cases, in secretory vesicles) via HS proteoglycans (HSPGs) [100]. As stated above, they are essential co-receptors to most FGFs, binding both the ligand and the receptor to mediate signal complex formation. In addition, they shield FGF ligands from enzymatic degradation, can establish FGF ligand reservoirs in the ECM and regulate their diffusion and endocytosis [101]. They do not seem to be essential for the direct ligand:receptor inteactions of BMPs, but they nonetheless modulate BMP signaling in various ways: They may increase local ligand concentrations, facilitate short distance diffusion (by repeated dissociating and re-associating) or trap BMP antagonists, and thereby enhance BMP signals; or they may reduce such signals by immobilizing ligands or antagonist-ligand pairs or by elevating ligand clearance via endocytosis [100,102]. All these properties make HSPGs indispensable for gradient formation of both FGFs and BMPs and hence their morphogen functions. HSPGs modulate the speed and the range of ligand movement in the ECM, depending on their type and their modifications and on the excess of HSPG shedding (the cleaving of ligand-binding HS fragments from the core protein) [101,103]. Unfortunately, competition of morphogens for HSPG binding in the context of gradient formation has been hardly studied, although one research team has suggested that spatiotemporally regulated modifications of HSPGs may alter ligand-specificity and thereby facilitate “morphogenic switches” [104]. If and how certain HSPG modifications influence BMP-FGF interaction by preferentially supporting signaling via one ligand family over the other remains to be elucidated.

## 5. Conclusions

If interactions between members of the FGF and BMP families are as diverse and context-dependent as described above, how do we approach finding more general connections between them?

To identify common mechanisms, combining models that reduce the number of influencing parameters seems to be a good start. In drosophila melanogaster, the growth factor families are much smaller, providing the complexity of an entire organism without that wide range of ligands and receptors. Cell culture models also provide a reduced set of ligands and receptors; and make spatiotemporal parameters both optional and controllable.

Further, mathematical models are a great tool to compare what is possible to what is observed. Before the nature of morphogens was even understood, such models already were potent tools to describe morphogen gradients as basis for the formation of complex anatomy [105]. A more recent example looks at FGF/BMP synergy in the context of the formation of digits. Indeed, the model was only able to accurately predict digit patterning, as observed in healthy and mutant mice, if BMP and FGF positively influenced each other’s levels of concentration [106]. FGF/BMP antagonism has likewise been modelled. In murine brain development, FGF2/8 and BMP4 form opposing shallow gradients that nevertheless result in a sharp tissue border. By linking observed cell behavior with mathematical models premising inhibition, a mode of cross-inhibition was proposed that explained the cells’ hypersensitivity to BMP4 in dependence of FGF2, their BMP4 hysteresis and their individual thresholds (EC_50_ values) for the induction of different BMP4 targets: In a cross-inhibitory positive feedback loop, un-specified intermediates in both FGF and BMP signaling cross-inhibit each other, affecting downstream targets in both signaling cascades in dependence of their inhibitory effectiveness, their abundance, and their influence on said targets [62].

Certainly, there are examples where downstream components of both pathways are (at least unilaterally) antagonizing, as exemplified in the inactivation of R-SMADs by Erk-mediated linker-phosphorylation, or in the inhibition of bHLH factors downstream of FGF2 by ID proteins downstream of BMP4 [107]. However, more often than not, BMP-FGF interaction is linked by intermediates operating on the level of transcription. Certainly, this allows for a highly cell context-dependent result; and it would explain why synergistic and antagonistic effects of these two growth factor families co-exist in many tissues.

In this context, it is noteworthy that canonical signal transduction via SMADs does not include signal amplification by repeated phosphorylation steps, as is the case in MAPK signaling. On the contrary, SMADs, the agents that both transport and execute the signal inside of the nucleus, are also the ones directly activated by the receptor. Combined with the fast and constitutive dephosphorylation of SMADs in activated cells, this results in a linear, proportional relation of receptor activation and SMAD phosphorylation. Thus, BMP ligand-induced receptor activation does not “switch on” a response that is amplified and hence stabilized inside the cell, as is the case for FGF ligand-induced signals, but rather elicits a readily reversible activated cell state in close relation to BMP receptor activity [7]. These very different ways of signal transduction from underneath the cell surface to the nucleus further support that primary FGF-BMP interaction happens at the level of transcription.

An interesting additional possibility is the integration at the cell surface, as has been discussed above. This area of interaction has hardly been explored, but there are indications that it would deserve to be. Surely, one indicator is the observation that heparan sulfates play such a pivotal role in gradient formation of both BMPs and FGFs that their influence on morphogen crosstalk is far more likely than not. Another is the recent description of muscle-specific kinase MuSK as a BMP co-receptor [108]. MuSK is a receptor tyrosine kinase (RTK), just like FGF receptors, but apart from its genuine ligands, it can also bind to and positively influence signaling for BMPs 2, 4 and 7, independently of its own kinase activity. It is intriguing to imagine that more ligand-receptor pairs that span two different protein families may exist. Such cross-overs would open a new chapter of signaling crosstalk mechanisms that could potentially touch on more than just FGF-BMP interactions.
